# Medical Institute of Louisville

**Published:** 1844-03

**Authors:** 


### MEDICAL INSTITUTE OF LOUISVILLE.

Dr. Cooke, as announced in our last number, having resigned, the Managers of the Louisville Medical Institute have united the chairs of Theory and Practice, and Pathological Anatomy and Clinical

Medicine in one professorship, under the title of "*Pathology and Practice of Medicine,*" to which Dr. Drake has been appointed. The Institute has now the number of professors usual in the medical schools of this country.

At the late annual Commencement, the degree of *Doctor of Medicine* was conferred on forty-seven young gentlemen. The honorary degree of M. D. was conferred on Dr. Charles G. Ballard, of Green Castle, Indiana, and on Dr. Silas Ames, of Montgomery, Alabama. Y.

